# Limitations in activities of daily living and support needs – Analysis of GEDA 2019/2020-EHIS

**DOI:** 10.25646/9570

**Published:** 2022-03-30

**Authors:** Judith Fuchs, Beate Gaertner, Franziska Prütz

**Affiliations:** Robert Koch Institute, Berlin Department of Epidemiology and Health Monitoring

**Keywords:** ACTIVITIES OF DAILY LIVING, OLDER PERSONS, GERMANY, HEALTH MONITORING

## Abstract

Being able to perform activities of daily living is an important component of a person's ability to function. If these activities are impaired, support is needed. Using data from GEDA 2019/2020-EHIS, we present how many people aged 55 and older living in private households in Germany experience limitations in activities of daily living. Severe limitations in basic (fundamental) activities (e.g. food intake) are reported by 5.8% of women and 3.7% of men. The proportion increases with age as 13.4% of women and 9.0% of men aged 80 and older experience limitations. Severe limitations of instrumental activities of daily living (e.g. grocery shopping) are rather rare in participants less than 80 years of age. But at age 80 and older the proportion rises to 35.9% of women and 21.0% of men. A total of 68.1% of afflicted women and 57.5% of men receive help and support related to limitations of basic activities. Women are also more likely to report a lack of support (48.8% vs. 43.2%). The situation is slightly better with regard to instrumental activities.

The results of GEDA 2019/2020-EHIS show in which areas of daily life older and very old people are impaired, give an impression of who is affected particularly strongly and indicate where support services are insufficient. As such, these results provide clues as to where support can be provided to enable older people to keep living in their own homes for a long time.

## 1. Introduction

As a result of the ongoing demographic change, the proportion of the population accounted for by older people is increasing; according to the Federal Statistical Office, the number of people aged 67 and older in Germany will rise by 22% between 2020 and 2035 [1]. Although people age very differently, the likelihood of illness and declining physical and cognitive performance consistently increases with age [2]. The recording of limitations in basic and instrumental activities of daily living provides evidence as to where particular deficits exist and thus points to opportunities to improve the overall situation of older people [2–4].

The number of people reporting limitations in activities of daily living increases with age, and this holds true in Germany as well [5]. These limitations restrict people in their participation and autonomy and they are dependent on help. In the course of the ongoing demographic change, the number of people affected will continue to rise in the future.

**GEDA 2019/2020-EHIS**

Fifth follow-up survey of the German Health Update

**Data holder:** Robert Koch Institute

**Objectives:** Provision of reliable information on the health status, health behaviour and health care of the population living in Germany, with the possibility of European comparisons

**Study design**: Cross-sectional telephone survey

**Population:** German-speaking population aged 15 and older living in private households that can be reached via landline or mobile phone

**Sampling:** Random sample of landline and mobile telephone numbers (dual-frame method) from the ADM sampling system (Arbeitskreis Deutscher Markt- und Sozialforschungsinstitute e.V.)

**Sample size:** 23,001 respondents

**Study period:** April 2019 to September 2020

**GEDA survey waves:**

► GEDA 2009
► GEDA 2010
► GEDA 2012
► GEDA 2014/2015-EHIS
► GEDA 2019/2020-EHIS

Further information in German is available at www.geda-studie.de

It is unclear how many people in the general population aged 55 and older currently living in Germany experience limitations in activities of daily living, which areas are particularly limited, and which group of people lacks support with activities of daily living. Another matter of interest is a description of associations with other health indicators and sociodemographic variables [6].

The German Health Update (GEDA) surveys activities related to personal care and household activities. The aim of the present paper is to describe the presence of limitations of activities of daily living (Info box) among people aged 55 and older in Germany by gender and age group. In addition, a characterisation of impaired and unimpaired participants by disease-relevant and sociodemographic characteristics is presented here. It will also be shown whether or not impaired persons receive sufficient help. This serves to identify participants who are clearly afflicted by limitations and to illustrate prevention potentials and health care needs.

Self-assessed health status is an indicator that reflects the perception of one’s own health, encompassing not only physical health but also psychological status and quality of life [7]. Analyses related to limitations of activities show that the self-assessed health status is a predictor of ensuing limitations [8].

Health-related limitations in daily living are captured by the Global Activity Limitation Indicator (GALI), which uses the International Classification of Functioning, Disability and Health (ICF) [4] as a conceptual framework and functions as a global, self-reported measure of the limitation of participation [9].

There is a significant correlation of visual and hearing impairments and limitations in activities of daily living, with no gender differences found. Early detection and effective treatment of visual and hearing impairments are important to prevent limitations in activities of daily living and to improve the independence in older people [10]. Mobility limitations are also often preceded by limitations in basic (fundamental) and instrumental activities of daily living (ADL/IADL limitations) and can thus serve as a clue for preventive measures [11].

Among the possible sociodemographic influencing factors, in addition to age, gender plays a central role for overall health and thus also for the ADL/IADL status [12]. Low education and poverty are risk factors for limitations of ADL and IADL [13, 14]. In addition, family composition also has a significant influence, as shown by results from the Irish longitudinal study [15]. It is known from the USA and from the SHARE study that urban and rural regions differ in the frequency of limitations [16, 17].

## 2. Methodology

### 2.1 Study design and sampling

GEDA is a nationwide cross-sectional survey of the resident population living in Germany (Info box). The GEDA survey has been conducted by the Robert Koch Institute (RKI) on behalf of the Federal Ministry of Health at multi-year intervals since 2008 and is a component of health monitoring at the RKI [21, 22]. The fifth follow-up survey, GEDA 2019/2020-EHIS, took place between April 2019 and September 2020 using computer-assisted, fully-structured interviews over the phone. The survey was based on a random sample of landline and mobile phone numbers (dual-frame method) [23]. The population comprised the population aged 15 and over living in private households whose usual place of residence at the time of data collection was in Germany. A total of 23,001 individuals with usable interviews participated in GEDA 2019/2020-EHIS (12,101 women, 10,838 men, 62 of other gender identity or no information provided). The response rate according to the standards of the American Association for Public Opinion Research was 21.6% [24]. A detailed description of the methodology as well as of the classification of the response rate of GEDA 2019/2020-EHIS is available elsewhere [25]. Questions concerning limitations in activities of daily living were asked only after age 55, so the present sample includes 12,985 persons (7,086 women, 5,871 men, 28 of other gender identity or no information provided).

**Info box Basic and instrumental activities of daily living (ADL/IADL)**

According to the International Classification of Functioning, Disability and Health (ICF), an activity impairment is a difficulty or inability a person may have in performing a particular activity.

In research and practice, the recording of limitations in activities of daily living is often done with the help of two instruments that record limitations in the so-called basic activities (activities of daily living, ADL) and the instrumental activities of daily living (instrumental activities of daily living, IADL). ADLs include the basic activities of meeting basic needs, such as eating, personal hygiene, getting up, dressing, or using the toilet. The most commonly used indices were published by Katz et al. [18] in 1963 and by Mahoney and Barthel [19] in 1965. IADLs include more elaborate tasks of daily living that are more complex to accomplish. These include, for example, activities such as making telephone calls, shopping, doing banking, housekeeping, taking medications, and using transportation. IADL are captured using a score based on the work of Lawton and Brody from 1969 [20].

ADLs are assessed in GEDA 2019/2020-EHIS via the variables of feeding, getting in and out of a bed or chair, dressing and undressing, using toilets, and bathing or showering (according to Katz et al. 1963). IADLs are assessed by means of the following activities: Preparing meals, using the telephone, shopping, managing medication (e.g. preparing pillboxes), doing light housework (e.g. washing dishes), doing occasional heavy housework (e.g. mopping floors), and taking care of finances and everyday administrative tasks (e.g. paying bills) (according to Lawton and Brody 1969).

Source: Adapted from Gaertner et al. 2019 [5]

### 2.2 Indicators

#### Limitations in activities of daily living

Internationally established instruments of the European Health Interview Survey (EHIS) were used to assess the limitations in activities of daily living in everyday life [26]. The questions measure the capability and the help received or needed in relation to five basic activities (ADL) according to Katz et al. [18] and seven instrumental activities of daily living (IADL) according to Lawton and Brody [20] (Info box). Participants were asked whether they would normally have difficulty doing that activity without help. The response categories were ‘No difficulty’, ‘Some difficulty’, ‘A lot of difficulty’, and ‘Cannot do at all/Unable to do’. The IADL included ‘Not applicable (I have never tried or done)’ as an additional response category. For the analyses concerning existing limitations, the variables were dichotomised: ‘A lot of difficulty/Cannot do at all’ versus ‘No/some difficulty/not applicable’. On this basis, the variables on the respective ADL and IADL limitations were generated. Participants who reported at least one ADL or IADL limitation were defined as ADL- or IADL-limited.

Participants with an ADL and/or IADL limitation were asked the following question to analyse the level of help received: ‘Thinking about all personal care/household activities where you have difficulty in doing them without help. Do you usually have help with any of these activities?’ with response options of ‘Yes, with at least one activity’ and ‘No’. The help received in each case was coded ‘Yes’ or ‘No’. Another question asked individuals with help if more help was needed and individuals without help were asked if help was needed. By definition, ‘(More) help needed’ was evident when more help or any help was needed according to the self-assessment.

#### Covariates

The three questions of the Minimum European Health Module (MEHM) [27] summarise the self-assessment of general health, the presence of chronic diseases, and the health-related limitations on daily living. The MEHM is part of the European Survey on Income and Living Conditions (EU-SILC) and of the EHIS and provides comparable information on the subjective perception of one’s own state of health across Europe.

The self-assessed general health status is recorded according to a recommendation of the World Health Organization (WHO) using the following question: ‘How is your health in general?’ The surveyed participants were asked to select one of five given response options. For the evaluation, these were dichotomised, whereby: ‘Very good’, ‘Good’, ‘Fair’ versus ‘Bad’, ‘Very bad’ were combined [27]. The presence of a chronic disease or a long-standing health problem was recorded using the following question: ‘Do you have any long-standing illness or health problem? This refers to illnesses or health problems that lasted, or are expected to last for 6 months or more’. Response options were ‘Yes’, ‘No’, or ‘Don’t know’.

Health-related limitations on daily living were recorded using the Global Activity Limitation Indicator (GALI) via respondent self-report [27]. The question was ‘Are you limited by a health problem in activities people usually do?’ (response categories: severely limited, limited, but not severely, not limited at all). Participants with limitations were additionally asked ’Have you been limited at least the past 6 months?’ (response categories yes and no). The period of ‘At least 6 months’ was developed at European level to take account of the presence of a long-term limitation [28]. This concept was adopted for the analyses; participants who had been limited for more than six months are defined as having longer-term health limitations. All other participants are considered to have no long-term limitations.

Vision impairment was recorded as follows: ‘Do you have difficulty seeing even when wearing your glasses or contact lenses? Would you say... none, some, a lot of difficulty, or cannot do at all/unable to do’. These were dichotomised for the analyses: no severe difficulties (none and some difficulties) and severe difficulties (a lot of difficulties or cannot do at all).

Impaired hearing was recorded through two questions: ‘Do you have difficulty hearing what is said in a conversation with one other person in a quiet room, even when using your hearing aid?’ and ‘Do you have difficulty hearing what is said in a conversation with one other person in a noisier room, even when using your hearing aid?’ each with response options of: ‘Would you say... none, some, a lot of difficulty, or cannot do at all/unable to do’. For the analyses, these were summarised into a dichotomous variable as difficulties in hearing: no serious difficulties (no or some difficulties in each case) and serious difficulties (at least once a lot of difficulties or cannot do at all).

Mobility limitations were assessed with the questions: ‘Do you have difficulty walking half a kilometre, or 500 meters, on level ground without the use of any aid?’ and ‘Do you have difficulty walking up or down 12 steps? Would you say… no, some, a lot of difficulty or cannot do at all/unable to do’. For the analyses, these were summarised into a dichotomous variable as mobility limitations: no serious difficulties (no or some difficulties in each case) and serious difficulties (at least once a lot of difficulties or cannot do at all).

Gender identity was used to describe gender differences. Participants could indicate which gender they felt they belonged to (female, male, other gender identity). Due to the small number of cases, participants who indicated a different gender identity or no gender identity are not shown in the analyses by gender. For the analyses, age in years was divided into age groups 55 to 64, 65 to 79, and 80 years and older. For household size a dichotomous variable was created: a) Participants who reported living in a single-person household and b) participants who reported living in a multi-person household, regardless of household type (couple with or without children, single parent, etc.). Education levels were assigned to low, medium, and high education groups according to the CASMIN (Comparative Analyses of Social Mobility in Industrial Nations) classification using school and vocational educational attainment [29]. For income, the imputed equivalised income (income weighted by household size and composition, missing information is estimated) was used and participants with less than 60% of the median income were considered to be at risk of poverty. For municipality size, the political municipality size class (categorized as of: 31.12.2018) was used as the variable, divided into four categories: rural (population <5,000), small town (population 5,000–20,000), medium town (population 20,000–<100,000), and city (population 100,000 and more).

### 2.3 Statistical analysis

Prevalences are presented overall or stratified by gender identity, age and education level with 95% confidence intervals (95% CI). Prevalences are estimates of the proportion of participants in the target group affected at some point in time. Their precision can be assessed using confidence intervals – wide confidence intervals indicate greater statistical uncertainty in the results.

The analyses were performed applying a weighting factor in order to correct for deviations of the sample from the population structure. As part of the data weighting, a design weighting was first performed for the different selection probabilities (mobile and landline network). Subsequently, an adjustment was made to the official population figures related to age, sex, federal state and type of district (as of 31.12.2019). In addition, the sample was adjusted to the education distribution in the 2017 Microcensus according to the International Standard Classification of Education (ISCED classification) [30].

All analyses were conducted using Stata 17.0 (Stata Corp., College Station, TX, USA, 2017). In order to take the weighting appropriately into account when calculating confidence intervals and p-values, all analyses were calculated using the survey procedures of Stata 17.0. A difference between groups is assumed to be statistically significant if the corresponding p-value (‘Pearson 2 statistic for two-way tables’, i.e. Pearson’s chi^2^ statistic) is less than 0.05.

## 3. Results

### 3.1 Limitations in basic activities of daily living (ADL)

Individual ADL limitations were seldomly reported by women and men overall (0.3% to 4.5%, Annex Table 1). Regarding individual limitations, women and men aged 80 and older were significantly more likely to report difficulty bathing or showering (11.1% and 7.1%, respectively) and getting in and out of a bed or chair (4.6% and 4.4%, respectively) compared with those aged younger than 80.

**Figure 1:**
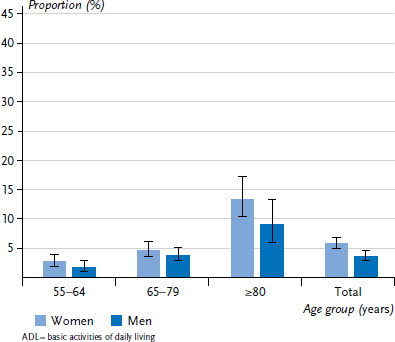
Proportion of participants reporting at least one severe ADL limitation by gender and age (weighted analyses) Source: GEDA 2019/2020-EHIS

The proportion of participants with severe limitations in at least one ADL was low, at 5.8% in women and 3.7% in men. There was a significant increase with age to 13.4% in women and 9.0% in men aged 80 years and older (Figure 1).

### 3.2 Limitations in instrumental activities of daily living (IADL)

Overall, the youngest age group experiences IADL limitations relatively rarely. All limitations show an increase in incidence with increasing age. The most frequently mentioned limitation is ‘doing occasional heavy housework’. It is reported overall by 13.9% of women and 7.9% of men, with women (33.5%) and men (19.6%) in the 80 years and older age group reporting it significantly more often (Annex Table 2). In second place, with a prevalence of 7.6% in women and 3.9% in men, is ‘shopping’, again more commonly among the very old (women 19.6%, men 9.1%). In third place is ‘taking care of finances and everyday administrative tasks’ (3.1% of women, 2.3% of men). Using the telephone causes problems for only a very small number of participants, which may also be explained by the survey mode (telephone interview).

**Figure 2:**
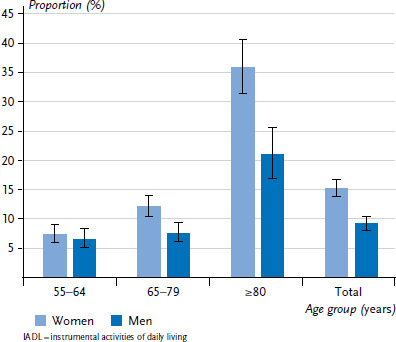
Proportion of participants reporting at least one severe IADL limitation by gender and age (weighted analyses) Source: GEDA 2019/2020-EHIS

Similar to ADL, the proportion of participants reporting at least one severe IADL limitation is rather low among those under 80 years of age. However, the proportion increases significantly among those aged 80 and older, at 35.9% of women and 21.0% of men in this age group (Figure 2).

### 3.3 Characterisation of groups of participants with limitations in basic and instrumental daily activities

**Figure 3:**
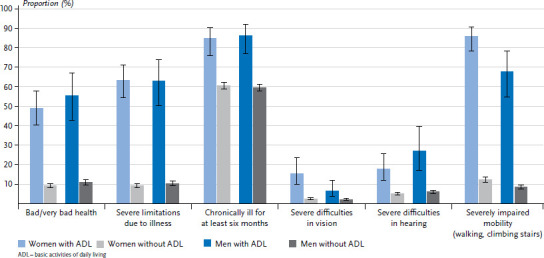
Proportion of health indicators by gender and ADL limitation (weighted analyses) Source: GEDA 2019/2020-EHIS

**Figure 4:**
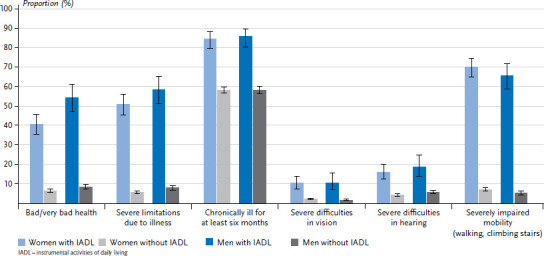
Proportion of health indicators by gender and IADL limitation (weighted analyses) Source: GEDA 2019/2020-EHIS

In the following, the results of the comparison of participants with and without limitations in basic and instrumental daily activities are presented with regard to the health indicators. It is evident for both genders that ADL- or IADL-limited are significantly more likely to report limitations in health status, health-related limitations (Global Activity Limitation Indicator, GALI), the presence of chronic diseases, vision and hearing, and mobility (Figure 3 and Annex Table 3). About one in two with ADL limitation (49.0% of women, 55.4% of men) report poor or very poor health, compared with about one in ten participants without ADL limitation (9.2% and 11.1%, respectively). The results for IADL limitations are similar (Figure 4 and Annex Table 3). Participants limited in their daily activities differ even more significantly with respect to health-related limitations (GALI): 63.3% of women and 63.0% of men with ADL limitation and 50.6% of women and 58.2% of men with IADL limitation report health-related limitations. By comparison, only about one in ten of those without ADL or IADL limitations report health-related limitations.

The majority of participants with ADL/IADL limitations have been chronically ill for at least six months: 84.8% of women and 86.3% of men with ADL limitation and 84.1% of women and 85.4% of men with IADL limitation (Figure 3, Figure 4 and Annex Table 3). In contrast, 60% of those without ADL/IADL limitation report being chronically ill.

Vision or hearing impairments are reported by few participants in the population aged 55 and older, but occur significantly more frequently among participants with ADL/IADL limitations (Annex Table 3). The difference is particularly marked for mobility limitations: About two-thirds of ADL-/IADL-limited women and men report them; especially women with ADL limitation (85.8%).

The sociodemographic data show that ADL- and IADL-limited participants are more likely to have a low level of education and a lower income and are more likely to live in single-person households than non-impaired participants (Annex Table 4). Among participants with ADL limitation, 58.8% of women and 61.0% of men have a low education level and 4.3% and 10.6%, respectively, have a high education level; among persons without ADL limitation, 43.4% of women and 42.1% of men have a low education level and 10.9% and 21.1%, respectively, have a high education level. Among participants with IADL limitation, 61.0% of women and 52.0% of men have a low education level and 5.0% and 13.1%, respectively, have a high education level; compared to 41.2% of women and 42.1% of men, respectively, and 11.5% and 21.5%, respectively, among persons without IADL limitation. A total of 30.6% of women and 29.3% of men with ADL limitations, but only 18.7% of women and 15.4% of men without ADL limitations live in poverty. Similar results are seen for IADL limitations. Participants with ADL or IADL limitation are more likely to live alone than participants without limitations: for women, the proportion is almost three-quarters; for men, the proportion is around 60% each, while only about half of women and about 40% of men without limitations live alone. There are no differences with regard to community size (urban/rural).

### 3.4 Support received and lack of support in performing ADLs and IADLs

The majority of participants with limitations in a basic activity of daily living (68.1% of women and 57.5% of men) (Table 1) indicate that they usually receive help with these activities. On average, women are more likely to receive help than men. However, the percentage of people who need (more) help varies between 35.0% and 53.7% depending on age group and gender.

With regard to help and support related to IADL limitation, it is evident that the majority of participants are not left to their own; 85.3% of women and 73.1% of men have people in their environment who provide help. However, again depending on gender and age group, every second or third person lacks the support they would need here (Table 1).

## 4. Discussion

The present results provide valid data on limitations in activities of daily living in a large sample of persons aged 55 years and older living in private households in Germany. The prevalence of limitations in ADL and IADL is generally low in Germany. About one in ten participants have IADL limitations, and a lower proportion report ADL limitations (5.8% of women, 3.7% of men). ADL and IADL limitations are associated to female gender, older age, lower education level, poorer health status, disease-related limitations, and impaired vision, hearing, and mobility. Results from the previous GEDA survey in 2014 [5] showed similar associations for Germany and for the countries of the European Union.

Women were found to be more likely to experience limitations than men in all three age groups, which is consistent with many European and non-European studies [31–33]. A Swedish study also shows that limitations tend to decrease across birth cohorts. However, it is not clear whether this is a real reduction or whether the limitations only occur later in life.

Limitations in ADL and IADL are usually due to existing chronic diseases, and the number of diseases and/or the presence of multimorbidity is another relevant factor [34]. Limitations in ADL and IADL arise in relation with (multi-)morbidity and IADL precedes ADL. The present results clearly show that ADL-limited participants are often impaired due to diseases.

Visual and hearing impairments are not very common in the population aged 55 years and older and seem to be compensated quite well by pertinent aids. These were included in the interview meaning that these limitations occur, possibly, with aiding devices. Again, it is evident that ADL- and IADL-limited participants are more likely to be afflicted, which may increase the risk of further loss of functional capacity [35].

People living alone are more likely to be limited in performing activities of daily living than people in multi-person households, which is consistent with other studies [36–38]. This has implications for policy and care. In this context, offers to support people living alone could possibly prevent more severe limitations if, for example, outreach assistance is made available.

An urban-rural difference with regard to the incidence of limitations, which was shown in one study [39] was not found in the present study. The GEDA data show no association between town/city size and proportions of ADL- or IADL-limited participants.

In addition, associations with socioeconomic status are evident: Participants with an ADL or IADL limitation are more likely to be at risk of poverty than individuals without an ADL or IADL limitation. Similar results are found, for example, in an English longitudinal study [40], which concluded that initiatives to improve social participation and social support for older people should be promoted. Especially with regard to support, which is lacking more often for impaired and very old people, there seems to be a need for improvement [41, 42]. Overall, it seems necessary to apply measures to reduce or reverse the limitations in activities of daily living of older people, for example by offering exercise programs or preventive home visits at the community level.

The need for help and support is differently well covered; those with limitations in basic activities receive help and support less frequently than those with limitations in instrumental activities. In addition, depending on age group and gender, approximately one-third to one-half of participants with limitations appear to lack support. This is consistent with findings from other studies [41, 43–45]. Informal helpers may less easily provide body-related support services than assistance with various household activities [46]. This should be considered for future assessments, for example in the context of a care needs assessment by the medical services of the health care insurance in the area of self-care with regard to the delivery of support.

As a limitation of the study, it should be noted that GEDA 2019/2020-EHIS is a general population-based cross-sectional study, based on telephone interviews in private households. Therefore, the available data do not allow a statement on the health status and functional limitations of nursing home residents. It can be assumed that the incidence of limitations among this population is higher than among people living in private households [46]. In addition, the data concerning severe hearing impairment in the general population, in particular, were probably underestimated in GEDA 2019/2020-EHIS, as these was a significant impediment to participation in a telephone survey. In addition, in these cases and also in the case of other factors impeding participation (e.g. speech disorders, cognitive limitations, or absences due to illness), a proxy interview was not conducted, so this may also have contributed to an underestimation of ADL and IADL limitations. Also, if there was only some difficulty in performing ADLs or IADLs, this was defined as no limitation in ADLs or IADLs. Methodological studies in this context should clarify the extent to which this definition is comparable in terms of the underlying competence dimensions relative to the other response categories.

Data collection took place from 2019 to 2020 and includes periods of strict containment measures during the COVID-19 pandemic. Analyses of changes in willingness to participate as a result are pending. However, analyses, for example, of changes in the need for support or assistance in the population aged 55 and older showed no pandemic-related variations [47]. Finally, the cross-sectional design does not allow any conclusions to be drawn about the causes, course or consequences of limitations of daily living.

Many studies also reported an association with cognitive functioning [15, 34]. Since this could not be adequately captured in GEDA 2019/2020-EHIS due to its procedure (telephone survey), no statements can be made in this regard. Further methodological studies are also needed for a more in-depth analysis of the gender differences described here as a function of gender roles, individual life situations and changes across birth cohorts.

The results of GEDA 2019/2020-EHIS show in which areas of daily life older and very old people are impaired, give an impression of who is affected particularly strongly and indicate where support services are insufficient. As such, these results provide clues as to where support can be provided to enable older people to keep living in their own homes for as long as possible.

## Key statements

- A total of 5.8% of women and 3.7% of men aged 55 and older are limited in at least one basic activity of daily living (ADL), whereby this proportion increases with age.
- The most common basic limitation of daily living reported by women and men of age 80 years and older is great difficulty in bathing or showering, at 11.1% and 7.1%, respectively.
- Only a small proportion of those under 80 experience limitations of instrumental activities of daily living (IADL), whereas 35.9% of women and 21.0% of men aged 80 and older experience these limitations.
- A total of 33.5% of women of age 80 and older and 19.6% of men of the same age report great difficulty in doing occasional heavy housework.
- Limitations of activities of daily living may be associated with being female, with older age, low education status, poor health, and impairments due to illness.

## Figures and Tables

**Table 1:** Proportion of participants with and without help for existing ADL and IADL limitations by gender and age (weighted analyses) Source: GEDA 2019/2020-EHIS

	Women			
	Age group (years)			
	55–64	65–79	≥80	Total
**ADL limitation**				
n	50	104	111	265
Help received (%) (95% Cl)	61.6 (42.4–77.8)	56.1 (41.3–69.9)	79.0 (64.9–88.4)	68.1 (59.0–76.0)
(More) help needed (%) (95% Cl)	53.7 (35.2–71.2)	50.2 (36.4–64.2)	46.0 (32.6–60.0)	48.8 (39.9–57.8)
**IADL limitation**				
n	160	308	310	778
Help received (%) (95% Cl)	79.3 (68.6–87.1)	80.9 (72.0–87.5)	90.6 (85.1–94.1)	85.3 (81.1–88.8)
(More) help needed (%) (95% Cl)	55.7 (44.5–66.3)	48.0 (39.6–56.6)	36.0 (28.2–44.5)	43.6 (38.3–49.1)

	Men			
	Age group (years)			
	55–64	65–79	≥80	Total
**ADL limitation**				
n	30	70	52	152
Help received (%) (95% Cl)	54.3 (28.4–78.1)	61.7 (44.3–76.5)	54.6 (32.2–75.3)	57.5 (44.7–69.3)
(More) help needed (%) (95% Cl)	35.0 (15.4–61.4)	54.0 (37.7–69.5)	35.6 (18.3–57.8)	43.2 (31.8–55.3)
**IADL limitation**				
n	105	160	147	412
Help received (%) (95% Cl)	66.7 (52.4–78.4)	74.5 (62.3–83.8)	77.5 (66.3–85.8)	73.1 (66.1–79.1)
(More) help needed (%) (95% Cl)	48.6 (35.0–62.5)	51.9 (30.9–63.6)	33.1 (23.1–44.9)	44.2 (37.1–51.6)

	Total			
	Age group (years)			
	55–64	65–79	≥80	Total
**ADL limitation**				
n	80	174	163	417
Help received (%) (95% Cl)	54.6 (38.3–70.0)	57.6 (46.3–68.1)	71.7 (58.8–81.8)	63.1 (55.5–70.0)
(More) help needed (%) (95% Cl)	50.3 (34.5–66.0)	51.0 (40.4–61.6)	42.9 (31.7–54.8)	47.4 (40.2–54.7)
**IADL limitation**				
n	265	468	457	1,190
Help received (%) (95% Cl)	74.3 (65.7–81.3)	78.7 (71.8–84.3)	87.1 (82.3–90.8)	81.4 (77.8–84.5)
(More) help needed (%) (95% Cl)	27.9 (24.0–32.3)	28.0 (24.7–31.5)	29.2 (25.0–33.8)	28.3 (26.1–30.7)

ADL = basic activities of daily living, IADL = instrumental activities of daily living, CI = confidence interval

## Appendix

**Annex Table 1:** Limitations in five basic activities of daily living by gender and age (percentage and confidence interval, weighted analyses) Source: GEDA 2019/2020-EHIS

	Women				Men				Total			
	Age group (years)				Age group (years)				Age group (years)			
	55–64	65–79	≥80	Total	55–64	65–79	≥80	Total	55–64	65–79	≥80	Total
**n**	2,756	3,303	1,027	7,086	2,365	2,734	772	5,871	5,121	6,037	1,799	12,957
**Proportion (%)**												
**Feeding yourself**	0.5	0.2	0.1	0.3	0.4	0.3	0.2	0.3	0.6	0.3	0.1	0.4
(95% Cl)	(0.1–1.6)	(0.1–0.8)	(0.0–0.3)	(0.1–0.7)	(0.1–1.8)	(0.1–0.9)	(0.1–0.6)	(0.1–0.8)	(0.3–1.5)	(0.1–0.6)	(0.1–0.3)	(0.2–0.7)
**Getting in and out of a bed or chair**	1.3	1.8	4.6	2.2	0.6	1.2	4.4	1.4	1.2	1.6	4.5	1.9
(95% Cl)	(0.7–2.4)	(1.2–2.7)	(2.9–7.2)	(1.7–2.9)	(0.3–1.2)	(0.7–2.1)	(2.1–8.8)	(1.0–2.2)	(0.7–1.9)	(1.1–2.1)	(3.0–6.6)	(1.5–2.4)
**Dressing and undressing**	1.6	1.5	3.1	1.9	0.7	1.8	3.4	1.6	1.4	1.6	3.2	1.8
(95% Cl)	(0.9–2.8)	(0.9–2.3)	(1.8–5.2)	(1.4–2.5)	(0.3–1.7)	(1.1–3.0)	(1.6–7.1)	(1.1–2.3)	(0.8–2.2)	(1.2–2.3)	(2.1–4.9)	(1.4–2.3)
**Using Toilets**	0.8	0.8	1,0	0.8	0.6	0.7	0.6	0.7	0.9	0.8	0.8	0.8
(95% Cl)	(0.3–1.8)	(0.5–1.3)	(0.4–2.2)	(0.5–1.2)	(0.2–1.6)	(0.4–1.5)	(0.2–1.6)	(0.4–1.1)	(0.5–1.7)	(0.5–1.2)	(0.4–1.5)	(0.6–1.2)
**Bathing or showering**	2.1	3.4	11.1	4.5	1.1	2.8	7.1	2.7	1.8	3.2	9.5	3.8
(95% Cl)	(1.3–3.2)	(2.4–4.8)	(8.3–14.7)	(3.7–5.5)	(0.5–2.3)	(1.9–3.9)	(4.5–11.0)	(2.0–3.5)	(1.2–2.7)	(2.5–4.0)	(7.5–12.1)	(3.2–4.4)

CI=confidence interval

**Annex Table 2:** Limitations in seven instrumental activities of daily living by gender and age (percentage and confidence interval, weighted analyses) Source: GEDA 2019/2020-EHIS

	Women				Men				Total			
	Age group (years)				Age group (years)				Age group (years)			
	55–64	65–79	≥80	Total	55–64	65–79	≥80	Total	55–64	65–79	≥80	Total
**n**	2,756	3,303	1,027	7,086	2,365	2,734	772	5,871	5,121	6,037	1,799	12,957
**Proportion (%)**												
**Preparing meals**	1.4	1.5	4.9	2.1	1.2	2.0	4.6	2.0	1.5	1.7	4.8	2.2
(95% Cl)	(0.8–2.2)	(0.9–2.5)	(3.2–7.4)	(1.6–2.8)	(0.6–2.4)	(1.3–3.0)	(2.9–7.4)	(1.5–2.7)	(1.0–2.3)	(1.2–2.4)	(3.5–6.5)	(1.8–2.7)
**Using the telephone**	0.5	0.1	0.6	0.3	0.0	0.1	0.2	0.1	0.2	0.1	0.5	0.2
(95% Cl)	(0.2–1.2)	(0.0–0.3)	(0.1–2.7)	(0.1–0.7)	–	(0.0–0.2)	(0.1–0.7)	(0.0–0.1)	(0.1–0.6)	(0.0–0.2)	(0.1–1.5)	(0.1–0.4)
**Shopping**	3.4	5.6	19.6	7.6	2.3	3.6	9.1	3.9	3.1	4.7	15.5	6.0
(95% Cl)	(2.4–4.9)	(4.4–7.1)	(15.7–24.1)	(6.5–8.9)	(1.4–3.8)	(2.5–5.2)	(6.3–13.0)	(3.1–4.9)	(2.3–4.1)	(3.8–5.7)	(12.8–18.6)	(5.2–6.8)
**Managing medication**	0.5	1.1	4.6	1.6	1.4	1.2	3.3	1.6	0.9	1.1	4.1	1.6
(95% Cl)	(0.2–1.0)	(0.6–2.2)	(2.8–7.5)	(1.1–2.3)	(0.7–2.9)	(0.6–2.2)	(1.5–6.7)	(1.1–2.4)	(0.5–1.7)	(0.7–1.8)	(2.7–6.1)	(1.2–2.1)
**Doing light housework**	2.2	2.4	8.4	3.6	1.5	3.2	6.4	2.9	1.9	2.8	7.6	3.3
(95% Cl)	(1.4–3.5)	(1.7–3.5)	(6.0–11.8)	(2.9–4.5)	(0.8–2.6)	(2.1–4.8)	(4.4–9.3)	(2.3–3.8)	(1.3–2.6)	(2.1–3.7)	(5.9–9.9)	(2.8–3.9)
**Doing occasional heavy housework**	6.4	11.4	33.5	13.9	5.1	6.7	19.6	7.9	6.0	9.2	28.2	11.2
(95% Cl)	(5.1–8.0)	(9.6–13.4)	(28.9–38.5)	(12.5–15.4)	(3.7–7.0)	(5.2–8.7)	(15.4–24.5)	(6.8–9.2)	(4.9–7.2)	(8.0–10.6)	(24.8–31.8)	(10.3–12.2)
**Taking care of finances and everyday administrative tasks**	0.9	1.6	10.1	3.1	2.1	1.5	5.2	2.3	1.7	1.6	8.2	2.8
(95% Cl)	(0.5–1.7)	(1.0–2.7)	(7.2–13.9)	(2.4–4.0)	(1.2–3.7)	(0.9–2.5)	(3.2–8.2)	(1.7–3.2)	(1.1–2.7)	(1.1–2.3)	(6.2–10.7)	(2.3–3.5)

CI=confidence interval

**Annex Table 3:** Basic and instrumental limitations of activities of daily living by gender and health-relevant limitations (percentage and confidence interval, weighted analyses) Source: GEDA 2019/2020-EHIS

	Women			Men			Total		
	With ADL	Without ADL	Total	With ADL	Without ADL	Total	With ADL	Without ADL	Total
**n**	265	6,821	7,086	152	5,719	5,871	417	12,540	12,957
**Proportion (%)**									
**Bad/very bad health status**	49.0	9.2	11.6	55.4	11.1	12.7	50.8	10.1	12.1
(95% Cl)	(40.2–58.0)	(8.1–10.5)	(10.3–12.9)	(42.9–67.3)	(9.6–12.7)	(11.2–14.3)	(43.5–58.0)	(9.2–11.1)	(11.1–13.1)
**Severe limitations due to illness**	63.3	9.2	12.3	63.0	10.5	12.5	61.9	9.9	12.4
(95% Cl)	(54.3–71.5)	(8.2–10.5)	(11.1–13.7)	(50.2–74.2)	(9.1–12.1)	(11.0–14.1)	(54.5–68.8)	(9.0–10.8)	(11.5–13.5)
**Chronically ill for at least six months**	84.8	60.6	62.0	86.3	59.6	60.5	84.1	60.1	61.3
(95% Cl)	(76.0–90.7)	(58.8–62.4)	(60.2–63.8)	(76.9–92.2)	(57.5–61.5)	(58.6–62.5)	(77.3–89.1)	(58.7–61.4)	(59.9–62.6)
**Severe difficulties in vision**	15.6	2.6	3.4	6.7	2.3	2.5	12.2	2.5	3.0
(95% Cl)	(9.8–23.9)	(2.0–3.4)	(2.7–4.3)	(3.6–12.2)	(1.7–3.1)	(1.9–3.3)	(8.2–17.8)	(2.1–3.0)	(2.5–3.5)
**Severe difficulties in hearing**	17.9	5.3	6.0	27.1	6.2	6.9	21.3	5.7	6.5
(95% Cl)	(11.9–26.0)	(4.4–6.3)	(5.1–7.1)	(17.1–40.1)	(5.2–7.3)	(5.9–8.1)	(15.8–28.1)	(5.1–6.5)	(5.8–7.3)
**Severely impaired mobility (walking, climbing stairs)**	85.8	12.4	16.6	67.9	8.5	10.7	79.9	10.5	14.0
(95% Cl)	(78.1–91.1)	(11.0–13.9)	(15.1–18.3)	(54.6–78.7)	(7.3–9.9)	(9.4–12.2)	(73.0–85.4)	(9.6–11.6)	(12.9–15.1)
	**With IADL**	**Without IADL**	**Total**	**With IADL**	**Without IADL**	**Total**	**With IADL**	**Without IADL**	**Total**
**n**	778	6,308	7,086	412	5,459	5,871	1,190	11,767	12,957
**Proportion (%)**									
**Bad/very bad health status**	40.4	6.4	11.6	54.2	8.5	12.7	44.8	7.4	12.1
(95% Cl)	(35.2–45.8)	(5.3–7.5)	(10.3–12.9)	(46.9–61.3)	(7.2–10.0)	(11.2–14.3)	(40.5–49.2)	(6.6–8.3)	(11.1–13.1)
**Severe limitations due to illness**	50.6	5.6	12.3	58.2	7.9	12.5	52.9	6.7	12.4
(95% Cl)	(45.1–56.1)	(4.7–6.6)	(11.1–13.7)	(50.9–65.2)	(6.6–9.4)	(11.0–14.1)	(48.4–57.2)	(5.9–7.6)	(11.5–13.5)
**Chronically ill for at least six months**	84.1	58.0	62.0	85.4	58.0	60.5	84.0	58.0	61.3
(95% Cl)	(79.3–88.0)	(56.2–59.9)	(60.2–63.8)	(80.0–89.6)	(55.9–60.1)	(58.6–62.5)	(82.2–87.1)	(56.6–59.4)	(59.9–62.6)
**Severe difficulties in vision**	10.3	2.2	3.4	10.5	1.7	2.5	10.3	1.9	3.0
(95% Cl)	(7.3–14.2)	(1.6–2.9)	(2.7–4.3)	(6.9–15.7)	(1.2–2.4)	(1.9–3.3)	(8.0–13.3)	(1.5–2.4)	(2.5–3.5)
**Severe difficulties in hearing**	15.9	4.2	6.0	18.7	5.8	6.9	16.7	5.0	6.5
(95% Cl)	(12.3–20.3)	(3.4–5.2)	(5.1–7.1)	(13.6–25.0)	(4.8–6.9)	(5.9–8.1)	(13.7–20.2)	(4.4–5.8)	(5.8–7.3)
**Severely impaired mobility (walking, climbing stairs)**	69.8	7.1	16.6	65.3	5.2	10.7	68.4	6.2	14.0
(95% Cl)	(64.6–74.5)	(6.0–8.4)	(15.1–18.3)	(58.4–71.6)	(4.2–6.4)	(9.4–12.2)	(64.3–72.2)	(5.4–7.0)	(12.9–15.1)

ADL = basic activities of daily living, IADL = instrumental activities of daily living, CI = confidence interval

**Annex Table 4:** Basic and instrumental limitations of activities of daily living by gender and sociodemographic parameters (percentage and confidence interval, weighted analyses) Source: GEDA 2019/2020-EHIS

	Women			Men			Total		
	With ADL	Without ADL	Total	With ADL	Without ADL	Total	With ADL	Without ADL	Total
**n**	265	6,821	7,086	152	5,719	5,871	417	12,540	12,957
**Proportion (%)**									
**Education level (CASMIN)**									
** Low education group**	58.8	43.4	44.3	66.2	42.1	43.0	62.3	42.8	43.7
(95% Cl)	(49.9–67.2)	(41.5–45.3)	(42.4–46.1)	(55.4–75.5)	(40.0–44.3)	(40.9–45.1)	(55.4–68.7)	(41.4–44.2)	(42.3–45.1)
** Medium education group**	36.8	45.8	45.3	23.2	36.8	36.3	31.3	41.6	41.1
(95% Cl)	(28.7–45.7)	(44.0–47.6)	(43.5–47.0)	(15.4–33.3)	(34.9–38.8)	(34.4–38.2)	(25.2–38.0)	(40.3–42.9)	(39.8–42.4)
** High education group**	4.3	10.9	10.5	10.6	21.1	20.7	6.4	15.6	15.2
(95% Cl)	(3.0–6.2)	(10.2–11.5)	(9.9–11.1)	(6.9–15.9)	(20.0–22.3)	(19.6–21.8)	(4.8–8.5)	(15.0–16.3)	(14.6–15.8)
**At risk of poverty**									
** <60% of median income**	30.6	18.7	19.4	29.3	15.4	15.9	31.7	17.3	18.0
(95% Cl)	(22.7–39.8)	(17.1–20.4)	(17.8–21.1)	(19.1–42.1)	(13.7–17.3)	(14.2–17.8)	(25.0–39.2)	(16.1–18.5)	(16.8–19.2)
**One-person household**									
** Yes**	71.6	53.0	54.0	62.9	42.6	43.3	69.2	48.1	49.2
(95% Cl)	(63.6–78.4)	(51.1–54.8)	(52.3–55.8)	(51.5–73.0)	(40.4–44.7)	(41.2–45.4)	(62.8–75.0)	(46.8–49.5)	(47.8–50.5)
**Size of municipality**									
** Rural**	7.7	11.2	11.0	6.6	12.2	12.0	7.2	11.7	11.5
(95% Cl)	(4.0–14.1)	(10.0–12.5)	(9.8–12.3)	(2.7–15.4)	(10.8–13.7)	(10.6–13.4)	(4.3–12.0)	(10.8–12.7)	(10.6–12.5)
** Small town**	26.4	27.0	26.9	26.6	29.0	28.9	26.3	27.9	27.8
(95% Cl)	(18.5–36.2)	(25.3–28.7)	(25.2–28.7)	(16.2–40.5)	(27.1–31.0)	(27.0–30.9)	(19.8–34.1)	(26.6–29.2)	(26.5–29.1)
** Medium town**	31.4	31.2	31.2	29.4	30.9	30.8	30.5	31.0	30.9
(95% Cl)	(23.3–40.8)	(29.4–32.9)	(29.5–32.9)	(18.8–42.8)	(28.9–32.9)	(28.9–32.8)	(23.8–38.0)	(29.7– 32.3)	(29.6– 32.3)
** City**	34.5	30.7	30.9	37.4	28.0	28.3	36.0	29.5	29.8
(95% Cl)	(26.9–43.0)	(29.1–32.4)	(29.3–32.6)	(26.7–49.5)	(26.3–29.8)	(26.6–30.1)	(29.7–42.9)	(28.3–30.7)	(28.6–31.0)
	**With IADL**	**Without IADL**	**Total**	**With IADL**	**Without IADL**	**Total**	**With IADL**	**Without IADL**	**Total**
**n**	778	6,308	7,086	412	5,459	5,871	1,190	11,767	12,957
**Proportion (%)**									
**Education level (CASMIN)**									
** Low education group**	61.0	41.2	44.3	52.0	42.1	43.0	58.3	41.7	43.7
(95% Cl)	(55.8–66.0)	(39.3–43.2)	(42.4–46.1)	(44.8–59.1)	(39.9–44.3)	(40.9–45.1)	(54.1–62.4)	(40.2–43.1)	(42.3–45.1)
** Medium education group**	33.9	47.3	45.3	34.9	36.5	36.3	34.0	42.1	41.1
(95% Cl)	(29.2–39.1)	(45.4–49.2)	(43.5–47.0)	(28.5–42.0)	(34.5–38.5)	(34.4–38.2)	(30.2–38.1)	(40.7–43.5)	(39.8–42.4)
** High education group**	5.0	11.5	10.5	13.1	21.5	20.7	7.7	16.2	15.2
(95% Cl)	(4.0–6.2)	(10.8–12.2)	(9.9–11.1)	(10.4–16.4)	(20.3–22.7)	(19.6–21.8)	(6.5–9.0)	(15.6–16.9)	(14.6–15.8)
**At risk of poverty**									
** <60% of median income**	28.9	17.7	19.4	30.6	14.5	15.9	30.0	16.2	18.0
(95% Cl)	(24.1–34.3)	(16.1–19.5)	(17.8–21.1)	(24.0–38.1)	(12.7–16.4)	(14.2–17.8)	(26.0–34.4)	(15.0–17.5)	(16.8–19.2)
**One-person household**									
** Yes**	72.0	50.8	54.0	60.9	41.5	43.3	68.5	46.4	49.2
(95% Cl)	(67.4–76.3)	(48.9–52.7)	(52.3–55.8)	(54.1–67.4)	(39.4–43.8)	(41.2–45.4)	(64.6–72.1)	(45.0–47.9)	(47.8–50.5)
**Size of municipality**									
** Rural**	8.0	11.5	11.0	7.3	12.4	12.0	7.8	12.0	11.5
(95% Cl)	(5.4–11.8)	(10.2–12.9)	(9.8–12.3)	(4.3–12.0)	(11.0–14.0)	(10.6–13.4)	(5.4–10.6)	(11.0–13.1)	(10.6–12.5)
** Small town**	28.1	26.7	26.9	19.9	29.8	28.9	25.3	28.1	27.8
(95% Cl)	(22.9–34.0)	(25.0–28.5)	(25.2–28.7)	(14.2–27.0)	(27.8–31.9)	(27.0–30.9)	(21.2–29.8)	(26.8–29.5)	(26.5–29.1)
** Medium town**	28.4	31.7	31.2	35.4	30.4	30.8	30.7	31.0	30.9
(95% Cl)	(23.6–33.6)	(29.9–33.5)	(29.5–32.9)	(28.6–42.9)	(28.4–32.5)	(28.9–32.8)	(26.7–35.0)	(29.6–32.4)	(29.6–32.3)
** City**	35.5	30.1	30.9	37.5	27.4	28.3	36.3	28.9	29.8
(95% Cl)	(30.5–40.9)	(28.4–31.8)	(29.3–32.6)	(30.6–44.9)	(25.7–29.2)	(26.6–30.1)	(32.1–40.6)	(27.7–30.1)	(28.6–31.0)

ADL = basic activities of daily living, IADL = instrumental activities of daily living, CASMIN = Comparative Analyses of Social Mobility in Industrial Nations, CI = confidence interval
